# Molecular Epidemiology of Enteric Adenovirus Gastroenteritis in under-Five-Year-Old Children in Iran

**DOI:** 10.1155/2016/2045697

**Published:** 2016-01-10

**Authors:** Anahita Sanaei Dashti, Pedram Ghahremani, Tayebeh Hashempoor, Abdollah Karimi

**Affiliations:** ^1^Shiraz HIV/AIDS Research Center, Shiraz University of Medical Sciences, Shiraz 7193613311, Iran; ^2^Pediatric Infectious Diseases Research Center, Shahid Beheshti University of Medical Sciences, Tehran, Iran; ^3^Professor Alborzi Clinical Microbiology Research Center, Namazi Hospital, Shiraz University of Medical Sciences, Shiraz 7193613311, Iran

## Abstract

*Background*. Acute gastroenteritis is one of the major sources of morbidity and mortality among young children in developed and developing countries. The aim of this study was to determine the prevalence of human adenovirus- (HAdV-) 40 and HAdV-41 in children hospitalized with gastroenteritis in five different health centers of Iran.* Methods*. In a cross-sectional epidemiological study, we studied 2682 fecal specimens that were collected from children under the age of 5 years in five educational and therapeutic pediatric centers in Iran from February 2012 to February 2013. Samples were tested for HAdV-40 and HAdV-41, using a specific pair of primers in polymerase chain reaction (PCR) method.* Results*. HAdV-40 and HAdV-41 were detected in 132 (5.18%) of the patients with diarrhea. A significantly higher prevalence of HAdV-40 and HAdV-41 (58.3%) was observed in children under 12 months of age, compared to other age groups. The male to female ratio was 1.7.* Conclusion*. The results of this study demonstrated that HAdV-40 and HAdV-41 could be considered etiological agents for acute gastroenteritis among children in Iran. The PCR as a rapid test may increase the chance for a relatively mild course of the disease followed by a complete recovery and avoiding administration of unnecessary antibiotics.

## 1. Introduction

Acute gastroenteritis is a very common disease that causes a significant mortality in developing countries. Worldwide, gastroenteritis affects 3 to 5 million children each year [1] and accounts for 1.5 to 2.5 million deaths per year or 12% of all deaths among children less than 5 years of age [1–3].

Though it seldom causes death in developed countries, it puts a heavy burden on the health care system [4], as it accounts for 10% of all hospital admissions for children under the age of 5 years.

Viruses are the most important etiologic causes responsible for approximately 70% of the episodes of acute gastroenteritis in children [4]. Worldwide, rotavirus is still the most common virus causing this disease [5, 6], followed by adenovirus types 40 and 41, astrovirus, and calicivirus [6, 7]. The rate of enteric adenovirus 40 and 41 varies from 1–8% in developed countries to 2–31% in developing countries [5, 7], but the prevalence is increased in immunocompromised patients [8].

Human adenoviruses (HAdVs) are one of the major causes of a number of different clinical syndromes including gastroenteritis, respiratory disease, conjunctivitis, hemorrhagic cystitis, and exanthema. They comprise 51 different serotypes (HAdV-1 to HAdV-51) grouped into 6 species, A to F. The enteric serotypes that are mostly associated with gastroenteritis are Ad-40 and Ad-41 which belong to species F.

Enteric adenoviruses associated with protracted diarrhea which may contribute to infant dehydration and malnutrition in developing countries [4, 5] spread predominantly by the fecal-oral route [3, 4]. Usually, after an incubation period of 8 to 10 days, periodic diarrhea occurs, with low grade fever, vomiting, abdominal pains, and dehydration [3, 6].

In a survey done in Taiwan, clinical features of enteric adenoviruses types 40 and 41 in children were diarrhea (96.9%), fever (54.7%), vomiting (45.3%), mild dehydration (43.8%), symptoms of upper respiratory tract infection (21.9%), and abdominal pain (12.5%) [9]. Long-lasting diarrhea (mean 10.8 days) was a predominant symptom of enteric adenoviruses in comparison to rotavirus [10]. In another comparative study, adenovirus gastroenteritis differed from rota- and astrovirus infections by subacute onset, less frequent vomiting, more frequent development of mild and moderate dehydration, and abdominal pains and distension [11].

These viruses exist year-round in all parts of the world but are most prevalent during spring, early summer, and midwinter in temperate climates [3, 5].

The present study was performed to identify the predominant adenovirus serotypes and their epidemiologic characteristics in infants and children with acute gastroenteritis from five different pediatric therapy centers in Iran.

## 2. Methods and Materials

The study was carried out from February 2012 to February 2013 on 2682 stool samples, obtained within 48 hours of admission to the hospitals of children under 5 years, suffering from acute gastroenteritis, from five different cities of Iran: Tehran, Shiraz, Mashhad, Tabriz, and Bandar Abbas. All the patients were examined according to the criteria described earlier for the number of episodes and the duration of vomiting and diarrhea, associated symptoms, and the extent of dehydration and treatment [12]. Patients with acquired or congenital immune deficiency were excluded from the study. The fecal specimens were stored at −70°C immediately for later virological tests. Informed consents were obtained from parents before collecting the samples. The Ethics Committee of Shahid Beheshti University of Medical Sciences approved the study. Demographic data were collected by questionnaires including sex, age, and living place. The specimens were stored at −80°C until processing to detect viral antigens.

The viral nucleic acid was extracted from stool suspensions by using the Bioneer kit, according to the manufacturer's instructions (Bioneer, Korea).

The extracted DNA was used as a template for amplification of HAdV-hexon gene. A 261 bp fragment was amplified using specific sense and antisense primers (Ref) including 5-GCCACCGATACGTACTTCAGCCTG-3 and 5-GGCAGTGCCGGAGTAGGGTTTAAA-3, respectively [13]. Briefly, the extracted DNA was amplified by standard PCR using primers recognizing the HAdV-hexon region. The reaction mixture consisted of 0.2 mM dNTP, 1.5 mM MgCl2, 1 U of Taq DNA polymerase (Fermentas, Lithuania), PCR-buffer 1x (Fermentas, Lithuania), and 1 mM of each primer. The PCR conditions consisted of 30 cycles of 4 min at 94°C, 30 s at 94°C, 62 s at 60°C, 60 s at 72°C, and a final extension cycle of 72°C for 7 min. PCR products were loaded on 1% agarose gel along with 100 bp molecular size DNA ladder (Fermentas, Lithuania).

Descriptive analysis including frequencies and nonparametric tests including chi-square and* t*-test were performed. Comparison of the mean values between groups was performed using the* t*-test. A *P* value of 0.05 or less was considered to be statistically significant. Data analysis was carried out using statistical software SPSS version 15.

## 3. Results

A total of 132 out of 2982 (5.18%) episodes of acute gastroenteritis were associated with Ad-40, Ad-41 genomic detection. The samples of healthy control were not positive.

**Figure 1:**
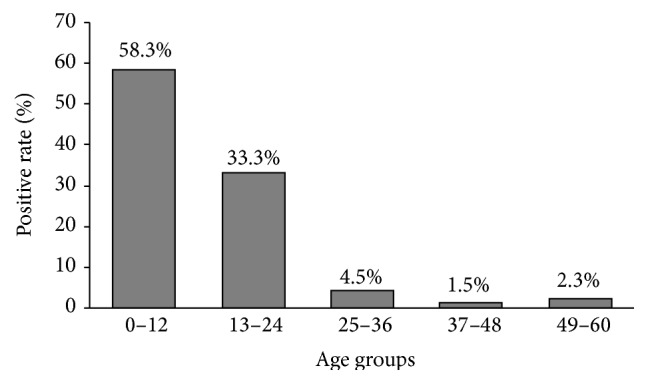
Age wise distribution of HAdV-40 and HAdV-41 positivity in gastroenteritis patients.

The highest incidence of diarrhea caused by Ad-40 and Ad-41 was in children less than 24 months of age (121 cases or 91.6%) and less than 12 months of age (77 cases or 58.3%) and in children between 6 and 12 months of age (32 cases or 24.2%) (Figure 1). The rate of infection was significantly different between children under 24 months of age and the older ones (Chi-Square, *P* < 0.0001). Males and females constituted 62.87% and 37.1% of the patients with positive result, respectively. The male to female ratio was 1.7.

## 4. Discussion 

Viruses are the most common cause of diarrhea in children. After rotavirus, enteric adenovirus is assumed as an important cause of viral diarrhea leading to noticeable deaths in children in many countries [14]. Among different types of HA, adenoviruses types 40 and 41 are one of the most common etiological agents of acute gastroenteritis among infants and young children less than two years of age [15, 16].

Fodha et al. examined 638 stool samples to assess the incidence of diarrhea related viral pathogens in Tunisian children. According to the results, rotaviruses, astroviruses, and adenoviruses types 40/41 were present in about 30% of specimen. The astroviruses had the highest incidence in diarrhea and the frequency of adenovirus strains was 6% in this study [17]. In a multicenter case-control study in Africa and south Asia on 9439 children, rotavirus and, to a lesser extent, adenovirus 40/41 (<5%) were the most common pathogens causing moderate-to-severe diarrhea in children [18].

Moyo et al. found that the frequency of adenovirus infection was higher in under-1-year-old infants. Also, they reported that nondiarrheic and diarrheic children had similar prevalence of adenovirus [19].

The other viruses that are now recognized to play a major role in sporadic gastrointestinal illnesses are noroviruses [20]. Early serosurveys documented a high prevalence of norovirus antibodies in children, but, because the virus was rarely detected in fecal specimens, its role in causing the infection seemed questionable [21].

The present study highlights the prevalence of enteric adenoviruses, HAdV-40 and HAdV-41, among acute gastroenteritis cases from five different cities of Iran. Viral DNA was detected using PCR technique. HAdV-40 and HAdV-41 infection was found in 5.18% of children with diarrhea but was not detected in the control group.

There are different reports about the prevalence of viral gastroenteritis in Iran. Previous epidemiological studies in Iran such as those done by Shokrollahi, Motamedifar, and Hamkar showed that 20%, 9%, and 2.3% of viral gastroenteritis were attributable to the adenoviruses, respectively [22–24]. However, a review of published data about viral gastroenteritis in Iran from 2002 to 2013 showed that enteric adenovirus incidence was 5.7% in children with acute gastroenteritis [25] which is consistent with our findings.

Also, a previous study conducted by Nakhaei Sistani et al. reported that 6.25% of Iranian children from neonates up to ten years old were positive for genomic HAdV-40 and HAdV-41 [26].

In other countries, a low rate of adenovirus infection was reported. In countries like Turkey, the USA, Thailand, Korea, the UK, Australia, Argentina, Sweden, and France, the prevalence of enteric adenovirus ranged from 1.55 to 15%, compared to that of our study, that is, 5.18% [27–32].

In an analysis of the age group with HAdV-40 and HAdV-41 infections, we observed that 91.7% (121/132) of positive cases were children under 2 years of age. The age distribution was similar to that reported by previous study [9].

In this study, vomiting, fever, abdominal pains, watery diarrhea, and dehydration were the most common specific symptoms in children with HAdV-40 and HAdV-41 infections, similar to previously reported study [33].

To avoid false-positive serological results caused by past infection of HAdV-40 and HAdV-41, using the species-specific PCR that is an available tool for the characterization of HAdV-40 and HAdV-41 with the advantage of testing clinical specimens directly is highly recommended.

There are some difficulties in establishing the diagnosis of HAdV-40 and HAdV-41 infections including the lack of a rapid and sensitive diagnostic method for use in public health laboratories and hospitals; consequently, the frequency of these viral infections is underestimated.

The main methods used to detect HAdVs in clinical samples such as blood and stools are antigen detection assays, such as enzyme-linked-immunosorbent assay (ELISA), immunofluorescence (IF) or immunochromatography tests, or PCR-based techniques. All immune-detection methods are of particular interest because they yield results quickly; besides, these methods are generally used for screening HAdVs in stool samples or respiratory fluids, since these kinds of samples are heavily loaded with HAdV particles, in case of infection [34, 35].

However, although the PCR test for diagnosis of these viral infections, HAdV-40 and HAdV-41, is the gold standard, it is not routinely performed on all stool specimens negative for bacterial and other viral infections including rotavirus, despite the fact that HAdV-40 and HAdV-41 are almost a common cause of infectious gastroenteritis.

Given such conditions, acute diarrhea remains a major public health problem in developing countries including Iran, and the detection of the HAdV-40 and HAdV-41 infections could improve the diagnostic coverage of viral gastroenteritis in children < 2 years of age, the major age group affected by this disease [15, 16].

## 5. Conclusion

Admitting the diarrhea as an important cause of mortality and morbidity in children worldwide and understanding the prevalence of pathogens involved in diarrhea are a key strategy for prevention policies and antibiotic stewardship programs. The present study showed that a small proportion of cases (5%) were attributable to adenoviruses types 40 and 41, a fact that would be leading in the above-mentioned issues.

## Abbreviations

HAdV: Human adenovirus
PCR: Polymerase chain reaction.
